# Computerized Clinical Decision Support System for Prompting Brief Alcohol Interventions with Treatment Seeking Smokers: A Sex-Based Secondary Analysis of a Cluster Randomized Trial

**DOI:** 10.3390/ijerph17031024

**Published:** 2020-02-06

**Authors:** Nadia Minian, Anna Ivanova, Sabrina Voci, Scott Veldhuizen, Laurie Zawertailo, Dolly Baliunas, Aliya Noormohamed, Norman Giesbrecht, Peter Selby

**Affiliations:** 1 Nicotine Dependence Service, Centre for Addiction and Mental Health, 175 College St, Toronto, ON M5T 1P7, Canada; nadia.minian2@camh.ca (N.M.); anna.ivanova@camh.ca (A.I.); sabrina.voci@camh.ca (S.V.); scott.veldhuizen@camh.ca (S.V.); laurie.zawertailo@camh.ca (L.Z.); dolly.baliunas@camh.ca (D.B.); aliya.noormohamed@camh.ca (A.N.); 2 Department of Family and Community Medicine, University of Toronto, 500 University Ave, Toronto, ON M5G 1V7, Canada; 3 Campbell Family Mental Health Research Institute, Centre for Addiction and Mental Health, 60 White Squirrel Way, Toronto, ON M6J 1H4, Canada; 4 Department of Pharmacology and Toxicology, University of Toronto, 1 King’s College Cir, Toronto, ON M5S 1A8, Canada; 5 Dalla Lana School of Public Health, University of Toronto, 155 College, Toronto, ON M5T 3M7, Canada; 6 Institute for Mental Health Policy Research, Centre for Addiction and Mental Health, 33 Russell St, Toronto, ON M5S 2S1, Canada; 7 Department of Psychiatry, University of Toronto, 250 College Street, Toronto, ON M5T 1R8, Canada

**Keywords:** alcohol, tobacco, smoking cessation, clinical decision support systems, brief intervention, sex differences

## Abstract

Although brief alcohol intervention can reduce alcohol use for both men and women, health care providers (HCPs) are less likely to discuss alcohol use or deliver brief intervention to women compared to men. This secondary analysis examined whether previously reported outcomes from a cluster randomized trial of a clinical decision support system (CDSS)—prompting delivery of a brief alcohol intervention (an educational alcohol resource) for patients drinking above cancer guidelines—were moderated by patients’ sex. Patients (*n* = 5702) enrolled in a smoking cessation program at primary care sites across Ontario, Canada, were randomized to either the intervention (CDSS) or control arm (no CDSS). Logistic generalized estimating equations models were fit for the primary and secondary outcome (HCP offer of resource and patient acceptance of resource, respectively). Previously reported results showed no difference between treatment arms in HCP offers of an educational alcohol resource to eligible patients, but there was increased acceptance of the alcohol resource among patients in the intervention arm. The results of this study showed that these CDSS intervention effects were not moderated by sex, and this can help inform the development of a scalable strategy to overcome gender disparities in alcohol intervention seen in other studies.

## 1. Introduction

Alcohol use is a leading cause of death and disability worldwide [1]. In Canada, 3.3 million persons drink alcohol at levels that put them at risk of immediate harm, such as injury, and 4.7 million drink at levels that put them at risk of developing chronic conditions such as cancer and liver disease [2,3]. Co-use of other substances with alcohol can further increase the risk of harm [4,5,6,7]. For example, co-use of tobacco with alcohol leads to a multiplicative increase in the risk of aero-digestive and other cancers [8,9,10,11]. 

There is substantial evidence that brief intervention in primary health care settings can reduce hazardous or harmful alcohol consumption in patients compared to minimal or no intervention [12]. However, fewer than one in four Canadians report discussing alcohol use with a health care provider (HCP) in the previous two years [13]. Despite recommendations to screen all adults in primary care for risky alcohol use [14,15], there is evidence that discussions about alcohol use and delivery of brief alcohol interventions vary by patient sociodemographic characteristics [16,17]. While brief intervention is effective for both men and women [12], HCPs are less likely to discuss alcohol use [17,18,19,20] or provide a brief alcohol intervention to women compared to men [16,21,22,23,24]. This might be due to the research showing that HCP decisions to provide a brief intervention are associated with the social acceptability of alcohol [25,26], and findings pointing out that drinking is seen as an appropriate masculine behavior [27] but which defies feminine stereotypes [28].

Although men generally consume more alcohol than women [2,29], women have higher levels of blood alcohol after drinking an equivalent amount of alcohol and are more vulnerable to many of the negative consequences of alcohol use [29,30]. In addition, there is evidence that harm due to the use of alcohol is increasing among women in Canada. The rate of alcohol-related deaths has increased among women in Canada by 26% since 2001, compared to an approximately 5% increase among men [31]. In Ontario, emergency room visits due to alcohol use increased 87% for women between 2003 and 2016, compared to an increase of 53% for men during the same period [32]. Thus, the need for intervention with women that consume risky levels of alcohol is critical.

Computer-based clinical decision support systems (CDSSs) are one promising method of promoting HCP adherence to recommended best practices [33,34]. A CDSS uses information technology to provide clinicians with patient-specific assessments or recommendations in a timely manner to assist with clinical decision-making, for example by providing real-time alerts and reminders [35]. There is evidence from research conducted internationally that CDSSs improve various provider behaviours including prescribing practices (e.g., adjustment of drug dose or duration) [36,37], performance of preventive services (e.g., vaccinations) [37,38,39], and ordering tests [37,40]. However, evidence for the positive impact of CDSSs on practice have been inconsistent [41] and sometimes small to modest in magnitude [37]. Studies with predominantly male, veteran samples in the United States have found that the results of an electronic clinical reminder on HCP delivery of a brief alcohol intervention are mixed, with either positive [42] or no effect [43].

We previously reported findings from the COMBAT study [44], a pragmatic cluster randomized trial conducted in 2016 to 2017 in Ontario, Canada, to test the effectiveness of a CDSS that prompted HCPs in primary care to deliver a brief alcohol intervention—providing an educational alcohol resource—with patients flagged for drinking alcohol above cancer guidelines [45]. The CDSS was integrated into a web-based assessment HCPs completed with patients enrolling in the Smoking Treatment for Ontario Patients (STOP) program, a smoking cessation program implemented in primary care clinics across Ontario that provides behavioural support and up to 26 weeks of nicotine replacement therapy to eligible patients at no cost. In the original sample, a total of 15,150 patients (99.6% of patients) were screened; 5715 patients were identified as drinking above the Canadian Cancer Society (CCS) guidelines; 2578 were offered an appropriate alcohol resource; and 483 accepted the resource. The CDSS prompt had no statistically significant effect on HCP offering of an educational alcohol resource but significantly increased patient acceptance of the resource, when offered, from 16% to 21% [44].

Health information technology has been raised as a potential means to promote health equity by encouraging more equitable treatment [46,47,48], and there is some evidence demonstrating its capability to reduce disparities in care [49,50]. As such, a CDSS prompt might be one tool with the potential to reduce disparities based on sex or gender in the assessment and management of alcohol use by HCPs. Previous efforts to implement a CDSS have had an inconsistent impact on sex and gender disparities. Integration of a CDSS into the electronic health record of an internal medicine practice did not result in equitable practice, as female patients were less likely to be screened for alcohol use compared to men, although they were not less likely to receive a brief intervention if they screened positive [51]. Another study found that implementation of a performance measure and clinical decision support tool enhanced sex disparities, such that implementation of the performance measure led to a greater increase in rates of brief intervention for men [24]. However, both of these studies had limitations due to the use of single cut-off score to identify risky alcohol use, rather than applying a lower cut-off score for women as recommended [52]. As such, it remains unclear whether the impact of CDSS implementation on HCP delivery of brief intervention for risky alcohol use is similar for men and women and whether a CDSS reduces disparities in brief intervention between men and women. Thus, the purpose of this secondary analysis was to examine whether the effect of a CDSS on HCP offering of an alcohol resource to patients or patient acceptance of the alcohol resource differed based on patient sex.

## 2. Materials and Methods 

### 2.1. Study Design and Sample

This was a sex-based, secondary analysis of the COMBAT study [44]. The patients in the trial were cigarette smokers who had enrolled in clinics across Ontario to obtain help with quitting smoking, who were also drinking above CCS guidelines [53]. Further trial eligibility criteria included enrolling in English and enrolling in person with their HCP completing the enrollment using the online portal designed for the program. As this secondary analysis was interested in the effects of patient sex on the main outcomes, the sample was further restricted to those who specified their sex as either male or female at enrollment, resulting in an analytic sample size of 5,702. Treatment arm randomization in the COMBAT study was performed at the site level to account for similarities among patient outcomes at the same clinic, as patients would have all been seen by the same HCP. Randomization was stratified by clinic type (family health team, community health center, nurse practitioner-led clinic) and predicted enrollment size at each clinic (i.e., small, large). Clinics were allocated on a 1:1 treatment versus control ratio to create balance between the two study arms. Further details about the cluster randomization can be found in previously published papers [44,53]. The COMBAT study was retrospectively registered at ClinicalTrials.gov, number NCT03108144, on 11 April 2017.

### 2.2. Study Variables

#### 2.2.1. Treatment Arms

Eligible patients enrolling at clinics in the treatment arm were screened for risky drinking based on CCS guidelines. If patients were found to be drinking at a level that exceeded the CCS safe drinking cut off (defined as having consumed in the previous week at least seven alcoholic beverages for women and at least 14 alcoholic beverages for men and/or any patient consuming 5 or more alcoholic beverages on a single occasion), the HCP conducting the enrollment received a computer generated prompt to provide a brief alcohol intervention and hand out an appropriate resource to address risky drinking behavior during that enrollment visit. The recommended brief intervention and resources differed depending on the severity of the patient’s risky drinking. More information about scoring of the CCS safe drinking cut off as well as descriptions of the brief interventions and resources offered by the HCPs in the study can be found in COMBAT’s protocol manuscript [53] and in the manuscript describing the design of the alcohol resources [54]. The HCPs of patients who did not exceed the CCS safe drinking cut off did not receive any prompting by the CDSS.

Patients enrolling at clinics allocated to the control arm were asked the same alcohol consumption screening questions but were not flagged for HCPs if they drank above the CCS safe guidelines. The HCPs were still able to provide a brief intervention and offer a resource to address risky drinking behavior, but the CDSS did not prompt them to do so and did not provide any guidance regarding which type of resource the HCP should offer. 

#### 2.2.2. Patients’ Sex

Patient sex was collected at baseline. As per STOP’s registration questionnaire, patients were asked to self-identify as male, female, or other. Only 15 patients (0.3%) in the COMBAT sample selected “other”. As this group lacked sufficient variability in outcomes and site level clustering variables for regression model convergence or detection of any differential effect, the analyses were limited to patients who self-identified as either male or female at enrollment.

#### 2.2.3. Outcomes

This analysis had two outcomes of interest, both recorded by the HCPs in the online enrollment portal: (1) the offer of an appropriate alcohol resource by the HCP and (2) the acceptance of the offered alcohol resource by the patient. The primary outcome, offer of an appropriate alcohol resource, was coded dichotomously, yes or no, for each eligible patient enrollment. An outcome of yes was defined as the HCP offering an alcohol reduction resource for patients who drank above the CCS guidelines but scored below 20 points on the AUDIT-10 [55], or an offer of an alcohol abstinence resource to patients who drank above CCS guidelines and also had an AUDIT-10 score of 20 points or more. An outcome of no appropriate offer made was defined as the HCP stating they will not offer an alcohol resource, or an offer of an inappropriate resource (i.e., an offer of a reduction resource to patients requiring an abstinence resource or vice versa).

The secondary outcome, acceptance of the alcohol resource by the patient, was also coded dichotomously, yes or no, for each eligible patient enrollment. An outcome of yes was defined as the HCP offering any alcohol education resource, and the patient not declining it. An outcome of no was defined as the patient declining the offered resource. Due to the functionality of the CDSS, an incorrect resource could only be offered to patients in the control arm, and the type of resource was recorded only after it was accepted. This outcome reflects the acceptance of any resource, regardless of appropriateness.

### 2.3. Statistical Analyses 

Descriptive statistics were used to describe males and females within each of the treatment arms on baseline characteristics including age, educational attainment, household income, employment status, smoking status, Heaviness of Smoking Index score [56], alcohol consumption, AUDIT-C [57] and AUDIT-10 scores, past year and lifetime attempts to quit smoking, recent marijuana and opioid use, and other health comorbidities. Descriptive statistics were also used to calculate crude primary and secondary outcomes within male and female subgroups of each treatment arm. To examine intervention and patients’ sex effects, three logistic generalized estimating equations (GEE) models were then fit for each outcome with clinical site as a cluster variable (with an exchangeable correlation matrix), robust standard errors, and the site stratification variables (i.e., site size and type) included as covariates. The first model tested the intervention effect and included treatment arm, clinic size, and clinic type which replicated the model presented in a previous manuscript reporting the study’s main findings [44]. Patient sex was added to a second model, to test for an overall sex difference. A treatment arm × sex interaction term was added to a third model, to test whether the intervention effect varied by sex. To better understand the range of likely true differences, we then calculated adjusted absolute differences and relative risks, along with 95% confidence intervals, for the differences among the treatment arm-specific sex effects.

With the exception of the addition of patient sex, the models were identical to those used in the original COMBAT analysis [44], as the goal of this work was to build on the previous analysis. As with the original COMBAT analysis, we adopted an intention to treat approach with clinics and patients analyzed in the originally assigned treatment arm. All analyses were conducted using Stata 14 [58].

### 2.4. Ethical Considerations

All patients gave their informed consent to participate in this research. The study was conducted in accordance with the Declaration of Helsinki and the COMBAT study protocol was reviewed and approved by the Research Ethics Board at the Centre for Addiction and Mental Health on 17 July 2015 (protocol number 035-2015).

## 3. Results

Baseline characteristics of our analytic sample by sex and treatment arm are presented in Table 1. There were some minor differences between males and females on several baseline characteristics. Specifically, the males in both treatment arms were slightly older, had lower rates of high school completion, were heavier smokers, had more lifetime quit attempts, and had greater rates of past 30 day marijuana use. Male patients in our sample also consumed more alcoholic beverages than females; however, this difference was due to the differential screen-in criteria for males versus females in the study. Overall, alcohol consumption and other Table 1 sex differences were similar between the intervention and control arms.

**Figure 1:**
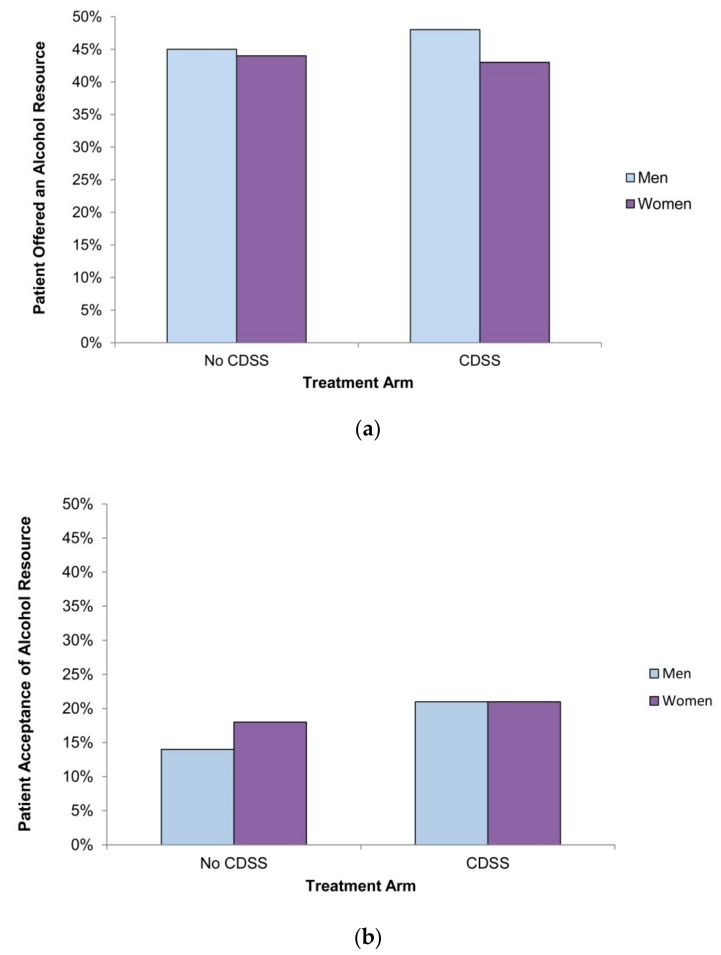
Proportion of men and women exceeding safe drinking guidelines (**a**) that were offered an appropriate educational alcohol resource by their health care provider in each treatment arm; (**b**) that accepted the offer of an educational alcohol resource by their health care provider in each treatment arm. CDSS = clinical decision support system.

Figure 1a,b show the crude rates of primary and secondary outcomes in each of the patients’ sex by treatment subgroups. In the control arm, 45% of males (702 patients) and 44% of females (548 patients) were offered an appropriate resource and 14% of males (100 patients) accepted the resource versus 18% of females (101 patients); in the intervention arm, 48% of males (760 patients) and 43% of females (560 patients) were offered an appropriate resource and 21% of males (157 patients) and 21% of females (120 patients) accepted it. Of the 201 accepted resources in the control arm, 45 (22%) were inappropriate, as defined by the study; 19% for females (19 of 101 patients) and 26% for males (26 of 100 patients), F(1,49) = 1.55, *p* = 0.22.

Results from the GEE analysis are presented in Table 2. The original model (presented in a previously published manuscript [44]) did not find implementation of the CDSS to be a significant predictor of patients exceeding safe drinking guidelines being offered an appropriate educational alcohol resource [44]. In the present analysis, patients’ sex was also non-significant as a covariate, and there was no evidence of a differential effect by sex.

The original model assessing the effect of the treatment arm on patient acceptance of the alcohol resource found that patients in the CDSS arm had significantly greater odds of accepting the alcohol resource when it was offered, compared to those in the control arm. The main effect of sex, when added, was not significant and neither was the sex × intervention interaction. There was therefore no evidence that the intervention effect with respect to resource acceptance differed by sex. Post-estimation calculations yielded an adjusted absolute difference in sex-specific intervention effects $\left(Mint-Mcontrol\right)-\left(Fint-Fcontrol\right)$ of 3.7% (95% CI = −1.9% to 9.3%) and a relative risk ratio $\frac{\left(Mint/Mcontrol\right)}{\left(Fint/Fcontrol\right)}$ of 1.27 (95% CI = 0.86 to 1.68). These confidence intervals indicate that any sex difference in the intervention effect is unlikely to be large, and suggest that the analysis was adequately powered to detect such a difference.

## 4. Discussion

The present study was a sex-based secondary analysis of the COMBAT study, a cluster randomized control trial which examined if the addition of a CDSS prompt influenced HCPs’ provision of a brief alcohol intervention to patients in a smoking cessation program who drank above recommended alcohol consumption guidelines, compared to HCPs who did not receive a CDSS prompt. The results of the COMBAT trial showed that the CDSS did not increase the likelihood of HCPs offering an educational alcohol resource; however, it did increase the likelihood of patients’ accepting the resource [44]. In this study, we found that these intervention effects were not moderated by sex.

The CDSS might have led to an increase in acceptance of alcohol resources among both men and women by influencing the way HCPs communicate with their patients. The CDSS provided guidance and concrete steps on what to do which might have made the resource more appealing to both male and female patients.

Less than half of eligible patients in the study were offered an alcohol resource, regardless of their sex or treatment arm, and less than a quarter of those offered an intervention actually accepted it, regardless of their sex. Prior research has shown similar or lower rates of alcohol intervention in primary care settings [42,59,60,61]. Thus, in this context—with this study showing that the CDSS led to an increase in the acceptance of alcohol resources and did not contribute to the sex inequities other interventions have shown [24]—the CDSS might be considered a success, as it led to more eligible patients receiving the alcohol resource.

Almost one quarter of resources accepted in the control arm conflicted with the study’s guidelines for what type of resource was appropriate (abstinence or reduction), but this was similar for males and females. The HCP may have deemed the resource appropriate for their patient based on other characteristics known about the patient that would influence the HCP’s judgment. For example, an HCP may choose to provide an alcohol reduction resource to a patient exceeding the AUDIT-10 cut off if they had intimate knowledge of the patient’s case and felt it unnecessary to offer an alcohol abstinence resource during that appointment.

Perhaps one reason we did not find a differential impact of the CDSS prompt on offering of the resource for males and females was due to the fact that we framed our study around cancer prevention, instead of alcohol abuse. Many researchers have pointed out that HCPs do not provide a brief alcohol intervention to their patients, especially female patients, due to fear of stigmatizing them [26,62,63,64]. Alcohol stigma attached to women is related to societal views of alcohol use being more masculine and women who drink not being able to be a good caretaker as expected [65]. Thus, framing the advice as a cancer prevention strategy might have felt less stigmatizing for the HCPs. Findings from other cancer prevention strategies, such as cancer screening or diet and physical activity advice, have found no sex- or gender-based disparities in HCPs recommendations [66,67]. 

These findings need to be understood in the general context where the intervention is taking place: a country where drinking among women has been increasing [2,68]; multi-million marketing campaigns encourage and normalize the use of alcohol, especially among women [69]; where alcohol has become increasingly accessible [70]; and where few Ontarians are aware of the association between alcohol drinking and cancer risks [45]. This is problematic given that researchers have found stronger alcohol control policies are associated with lower levels of alcohol consumption [71,72]. While a CDSS was not a good solution to prompt HCPs to deliver a brief alcohol intervention, without changing the social climate around alcohol and without policy interventions which have been shown to reduce alcohol consumption worldwide (such as tax increases [73,74,75,76,77,78], restricting setting use [72,78,79,80], and placing upper limits on the density of outlets [78,79,81]), it might continue to be hard to increase the proportion of males and females who receive an alcohol intervention beyond that achieved using the current CDSS.

The current study has several limitations. Although our analysis shows that large sex differences in the effectiveness of the CDSS are unlikely to be present, some caution is appropriate. Patients in the COMBAT study were asked to self-identify as male, female, or other; by not including specific questions about sex at birth and additional gender response options, this study is limited in its interpretability. Further, it is also possible that sex differences in intervention effects might be masked by sex differences in the severity of alcohol-related problems. This is a possibility we were able to address only partially via the testing of a possible effect for the AUDIT-C that was done during selection of the covariates for the original model published previously [44]. We did not ask participants to report if they were pregnant. While we presumed there were only a small number of pregnant women in our sample, it warrants examination in future studies, as this could have had an impact on our primary and secondary outcomes. For example, HCPs may have been motivated to offer an alcohol resource to pregnant patients due to the increased perception of risk, and pregnant women might have been more motivated to accept an alcohol intervention for similar reasons.

While a CDSS has the potential for reducing or removing disparities, it is important to note that in our sample, there was no apparent disparity in offering the alcohol resource in the control arm (without CDSS). Thus, it will be valuable to replicate this study in a setting where there is a gender disparity. Also important to note is that the CDSS was implemented in clinics that have a robust infrastructure for smoking cessation. All clinics had implemented the STOP program, which offers various supports such as a community of practice, an active listserv, several webinars a year on topics related to smoking cessation (including alcohol use), and ongoing operational support. These supports might make it hard to generalize these findings to other clinics that don’t have these supports.

Further research is needed to identify pragmatic implementation approaches that increase the delivery of brief alcohol interventions in primary care clinics. As other researchers have suggested, these might include the addition of a champion who can encourage HCPs to conduct a brief intervention to all patients who are drinking above guidelines and to problem solve barriers [74,82] as well as having ongoing training in alcohol screening and brief intervention. This might be particularly important in clinics with high staff turnover [82,83,84,85]. Both the champion and the training should emphasize the importance of making sure that sex and gender are taken into consideration when delivering brief alcohol intervention (e.g., addressing stigma and fear associated with reporting alcohol use during pregnancy and motherhood) [86,87,88,89]. Given that research has shown that the methods of program implementation can have a differential effect on males and females, it is critical to integrate a sex and gender lens to the conceptualization and evaluation of these approaches [90]. This will in turn ensure that more equitable health outcomes are achieved. In addition, future research should be conducted to understand the specific mechanisms through which the CDSS helped increase the proportion of patients’ acceptance of the alcohol resource, including an examination of whether there are different mechanisms for men and women.

## 5. Conclusions

We previously reported that implementation of a CDSS in primary care clinics across Ontario did not increase the offer of an alcohol resource to patients drinking above cancer guidelines but did increase acceptance of the resource. Given that implementation science as a field has been criticized for neglecting sex and gender considerations from its analysis [90] and widespread evidence of gender disparities in HCP delivery of brief interventions, it was important to examine whether sex moderated our results. The findings of this study suggest that CDSS effects were not moderated by patient sex. Regardless of patient sex, the CDSS prompt did not increase delivery of a brief alcohol intervention but did lead to increased acceptance of the resource. In increasing patient acceptance, the CDSS successfully increased the number of both male and female patients that ultimately received the alcohol resource that was appropriate. These findings can help inform future program and policy development needed to increase rates of brief alcohol intervention without creating, maintaining, or worsening any gender disparities in alcohol screening and intervention.

## Figures and Tables

**Table 1:** Baseline patient characteristics for the main analytic sample (*n* = 5702).

Variables	Control		Intervention	
	Male	Female	Male	Female
	(*n* = 1547)	(*n* = 1246)	(*n* = 1597)	(*n* = 1312)
Age in years (mean, SD)	49.1 (13.5)	46.9 (13.8)	48.5 (13.6)	46.8 (13.4)
Graduated high school	1033 (67)	977 (78)	1071 (68)	997 (76)
Household income above $40,000	532 (34)	373 (30)	495 (31)	372 (28)
Currently employed	827 (53)	660 (53)	829 (52)	693 (53)
Daily smoking status	1430 (92)	1177 (94)	1506 (94)	1238 (94)
Heaviness of smoking index > 3	417 (29)	229 (19)	441 (29)	273 (22)
Number of alcoholic drinks in past week (mean, SD)	12.9 (14.3)	7.6 (9.4)	12.5 (14.7)	8.2 (10.6)
Above AUDIT-C cut off	1270 (82)	1018 (82)	1290 (81)	1080 (82)
Above AUDIT-10 cut off	67 (4)	33 (3)	97 (6)	43 (3)
Past year attempts to quit smoking	797 (52)	631 (51)	808 (51)	685 (52)
Lifetime attempts to quit smoking ≥ 11	261 (17)	180 (14)	293 (18)	185 (14)
Marijuana use in past 30 days	546 (35)	303 (24)	599 (38)	367 (28)
Opioid use in past 30 days	244 (16)	175 (14)	244 (15)	189 (14)
Number of comorbid conditions endorsed ^1^ (mean, SD)	2.3 (2.0)	2.4 (2.0)	2.5 (2.1)	2.4 (2.0)

Note: Values are numbers (percentages) unless stated otherwise. SD = standard deviation. ^1^ Possible comorbid conditions (lifetime history of diagnosis) included: high blood pressure, high cholesterol, heart disease, stroke, diabetes, chronic bronchitis/emphysema/chronic obstructive pulmonary disease, rheumatoid arthritis, chronic pain, cancer, depression, anxiety, schizophrenia, bipolar disorder, substance use disorder, alcohol use disorder, or problem gambling.

**Table 2:** Adjusted odds ratios and 95% confidence intervals for offer and acceptance of an educational alcohol resource.

**CDSS as predictor of being OFFERED an appropriate alcohol resource *n* = 5702**						
	**Original model**		**With sex as covariate**		**With interaction term**	
	**OR (95% CI)**	***p*-value**	**OR (95% CI)**	***p*-value**	**OR (95% CI)**	***p*-value**
CDSS	1.20 (0.88–1.64)	0.25	1.20 (0.88–1.64)	0.25	1.24 (0.90–1.72)	0.19
Female patient	-	-	0.92 (0.82–1.03)	0.13	0.95 (0.82–1.11)	0.51
CDSS × Sex interaction term	-	-	-	-	0.93 (0.74–1.16)	0.53
**CDSS as a predictor of ACCEPTANCE of offered alcohol resource *n* = 2615**						
	**Original model**		**With sex as covariate**		**With interaction term**	
	**OR (95% CI)**	***p*-value**	**OR (95% CI)**	***p*-value**	**OR (95% CI)**	***p*-value**
CDSS	1.49 (1.01–2.18)	0.04	1.48 (1.01–2.16)	0.046	1.70 (1.12–2.57)	0.01
Female patient	-	-	1.12 (0.93–1.37)	0.24	1.33 (0.96–1.86)	0.09
CDSS × Sex interaction term	-	-	-	-	0.74 (0.50–1.09)	0.13

Note: Site stratification variables (i.e., clinic size and clinic type) were included as covariates.

## References

[1] Yasin Y.J., Banoub J.A.M. (2018). GBD 2016 Alcohol Collaborators. Alcohol use and burden for 195 countries and territories, 1990–2016: A systematic analysis for the Global Burden of Disease Study 2016. Lancet.

[2] Statistics Canada Canadian Tobacco, Alcohol and Drugs Survey (CTADS): Summary of Results for 2017. https://www.canada.ca/en/health-canada/services/canadian-tobacco-alcohol-drugs-survey/2017-summary.html.

[3] Young S.W., Candido E., Klein-Geltink J., Giesbrecht N. (2018). Preventing alcohol-related cancer: What if everyone drank within the guidelines?. Can. J. Public Health.

[4] Pennings E.J., Leccese A.P., Wolff F.A.D. (2002). Effects of concurrent use of alcohol and cocaine. Addiction.

[5] Chihuri S., Li G., Chen Q. (2017). Interaction of marijuana and alcohol on fatal motor vehicle crash risk: A case–control study. Inj. Epidemiol..

[6] Yurasek A.M., Aston E.R., Metrik J. (2017). Co-use of alcohol and cannabis: A review. Curr. Addict. Rep..

[7] Pennay A., Lubman D.I., Miller P. (2011). Combining energy drinks and alcohol: A recipe for trouble?. Aust. Fam. Physician.

[8] Franceschi S., Talamini R., Barra S., Barón A.E., Negri E., Bidoli E., Serraino D., La Vecchia C. (1990). Smoking and drinking in relation to cancers of the oral cavity, pharynx, larynx, and esophagus in northern Italy. Cancer Res..

[9] Pelucchi C., Gallus S., Garavello W., Bosetti C., La C.V. (2006). Cancer risk associated with alcohol and tobacco use: Focus on upper aero-digestive tract and liver. Alcohol Res. Health.

[10] Dawson D.A. (2000). Drinking as a risk factor for sustained smoking. Drug Alcohol Depend..

[11] Falk D.E., Yi H.-Y., Hiller-Sturmhöfel S. (2006). An epidemiologic analysis of co-occurring alcohol and tobacco use and disorders: Findings from the National Epidemiologic Survey on Alcohol and Related Conditions. Alcohol Res. Health.

[12] Kaner E.F., Beyer F.R., Muirhead C., Campbell F., Pienaar E.D., Bertholet N., Daeppen J.B., Saunders J.B., Burnand B. (2018). Effectiveness of brief alcohol interventions in primary care populations. Cochrane Database Syst. Rev..

[13] Canadian Institute of Health Information (CIHI) (2017). How Canada Compares: Results From The Commonwealth Fund’s 2016 International Health Policy Survey of Adults in 11 Countries—Accessible Report 2017.

[14] Curry S.J., Krist A.H., Owens D.K., Barry M.J., Caughey A.B., Davidson K.W., Doubeni C.A., Epling J.W., Kemper A.R., Kubik M. (2018). Screening and behavioral counseling interventions to reduce unhealthy alcohol use in adolescents and adults: US Preventive Services Task Force recommendation statement. JAMA.

[15] RNAO (2015). Engaging Clients Who Use Substances.

[16] Kaner E., Heather N., Brodie J., Lock C.A., McAvoy B.R. (2001). Patient and practitioner characteristics predict brief alcohol intervention in primary care. Br. J. Gen. Pract..

[17] McKnight-Eily L.R., Liu Y., Brewer R.D., Kanny D., Lu H., Denny C.H., Balluz L., Collins J. (2014). Vital signs: Communication between health professionals and their patients about alcohol use—44 states and the District of Columbia, 2011. Mmwr. Morb. Mortal. Wkly. Rep..

[18] Bertakis K.D., Azari R. (2007). Determinants of physician discussion regarding tobacco and alcohol abuse. J. Health Commun..

[19] Volk R.J., Steinbauer J.R., Cantor S.B. (1996). Patient factors influencing variation in the use of preventive interventions for alcohol abuse by primary care physicians. J. Stud. Alcohol.

[20] Denny C.H., Serdula M.K., Holtzman D., Nelson D.E. (2003). Physician advice about smoking and drinking: Are US adults being informed?. Am. J. Prev. Med..

[21] Angus C., Brown J., Beard E., Gillespie D., Buykx P., Kaner E.F., Michie S., Meier P. (2019). Socioeconomic inequalities in the delivery of brief interventions for smoking and excessive drinking: Findings from a cross-sectional household survey in England. BMJ Open.

[22] Bachrach R.L., Blosnich J.R., Williams E.C. (2018). Alcohol screening and brief intervention in a representative sample of veterans receiving primary care services. J. Subst. Abus. Treat..

[23] Lock C.A., Kaner E.F. (2004). Implementation of brief alcohol interventions by nurses in primary care: Do non-clinical factors influence practice?. Fam. Pract..

[24] Williams E.C., Lapham G.T., Rubinsky A.D., Chavez L.J., Berger D., Bradley K.A. (2017). Influence of a targeted performance measure for brief intervention on gender differences in receipt of brief intervention among patients with unhealthy alcohol use in the Veterans Health Administration. J. Subst. Abus. Treat..

[25] Rapley T., May C., Kaner E.F. (2006). Still a difficult business? Negotiating alcohol-related problems in general practice consultations. Soc. Sci. Med..

[26] Tam C.W.M., Zwar N., Markham R. (2013). Australian general practitioner perceptions of the detection and screening of at-risk drinking, and the role of the AUDIT-C: A qualitative study. Bmc Fam. Pract..

[27] Capraro R.L. (2000). Why college men drink: Alcohol, adventure, and the paradox of masculinity. J. Am. Coll. Health.

[28] Ricciardelli L.A., Connor J.P., Williams R.J., Young R.M. (2001). Gender stereotypes and drinking cognitions as indicators of moderate and high risk drinking among young women and men. Drug Alcohol Depend..

[29] Erol A., Karpyak V.M. (2015). Sex and gender-related differences in alcohol use and its consequences: Contemporary knowledge and future research considerations. Drug Alcohol Depend..

[30] National Institute on Alcohol Abuse and Alcoholism Are Women More Vulnerable to Alcohol’s Effects?. https://pubs.niaaa.nih.gov/publications/aa46.htm#:~:targetText=In%20general%2C%20women%20have%20less,the%20blood%20faster%20than%20men.

[31] Canadian Institute for Health Information Alcohol Harm on the Rise for Canadian Women. https://www.cihi.ca/en/alcohol-harm-on-the-rise-for-canadian-women.

[32] Myran D.T., Hsu A.T., Smith G., Tanuseputro P. (2019). Rates of emergency department visits attributable to alcohol use in Ontario from 2003 to 2016: A retrospective population-level study. CMAJ.

[33] Lobach D.F., Hammond W.E. (1997). Computerized decision support based on a clinical practice guideline improves compliance with care standards. Am. J. Med..

[34] Coma E., Medina M., Méndez L., Hermosilla E., Iglesias M., Olmos C., Calero S. (2019). Effectiveness of electronic point-of-care reminders versus monthly feedback to improve adherence to 10 clinical recommendations in primary care: A cluster randomized clinical trial. Bmc Med. Inform. Decis. Mak..

[35] Kawamoto K., Houlihan C.A., Balas E.A., Lobach D.F. (2005). Improving clinical practice using clinical decision support systems: A systematic review of trials to identify features critical to success. BMJ.

[36] Pearson S.-A., Moxey A., Robertson J., Hains I., Williamson M., Reeve J., Newby D. (2009). Do computerised clinical decision support systems for prescribing change practice? A systematic review of the literature (1990–2007). Bmc Health Serv. Res..

[37] Shojania K.G., Jennings A., Mayhew A., Ramsay C.R., Eccles M.P., Grimshaw J. (2009). The effects of on-screen, point of care computer reminders on processes and outcomes of care. Cochrane Database Syst. Rev..

[38] Bright T.J., Wong A., Dhurjati R., Bristow E., Bastian L., Coeytaux R.R., Samsa G., Hasselblad V., Williams J.W., Musty M.D. (2012). Effect of clinical decision-support systems: A systematic review. Ann. Intern. Med..

[39] Dexter P.R., Perkins S., Overhage J.M., Maharry K., Kohler R.B., McDonald C.J. (2001). A computerized reminder system to increase the use of preventive care for hospitalized patients. N. Engl. J. Med..

[40] Kastner M., Straus S.E. (2008). Clinical decision support tools for osteoporosis disease management: A systematic review of randomized controlled trials. J. Gen. Intern. Med..

[41] Groenhof T.K.J., Asselbergs F.W., Groenwold R.H., Grobbee D.E., Visseren F.L., Bots M.L. (2019). The effect of computerized decision support systems on cardiovascular risk factors: A systematic review and meta-analysis. BMC Med. Inform. Decis. Mak..

[42] Lapham G.T., Achtmeyer C.E., Williams E.C., Hawkins E.J., Kivlahan D.R., Bradley K.A. (2012). Increased documented brief alcohol interventions with a performance measure and electronic decision support. Med. Care.

[43] Williams E.C., Achtmeyer C.E., Kivlahan D.R., Greenberg D., Merrill J.O., Wickizer T.M., Koepsell T.D., Heagerty P.J., Bradley K.A. (2010). Evaluation of an electronic clinical reminder to facilitate brief alcohol-counseling interventions in primary care. J. Stud. Alcohol Drugs.

[44] Minian N., Baliunas D., Noormohamed A., Zawertailo L., Giesbrecht N., Hendershot C.S., Le Foll B., Rehm J., Samokhvalov A.V., Selby P.L. (2019). The effect of a clinical decision support system on prompting an intervention for risky alcohol use in a primary care smoking cessation program: A cluster randomized trial. Implement. Sci..

[45] Canadian Cancer Society The Truth About Alcohol. http://www.cancer.ca/en/about-us/news/on/2016/february/story4/?region=on.

[46] Pérez-Stable E.J., Jean-Francois B., Aklin C.F. (2019). Leveraging Advances in Technology to Promote Health Equity. Med. Care.

[47] US Department of Health and Human Services (2012). National Healthcare Disparities Report 2011.

[48] Graham G.N., Spengler R.F. (2009). Collaborating to end health disparities in our lifetime. Am. J. Public Health.

[49] Lee J. (2015). The impact of health information technology on disparity of process of care. Int. J. Equity Health.

[50] Lau B.D., Haider A.H., Streiff M.B., Lehmann C.U., Kraus P.S., Hobson D.B., Kraenzlin F.S., Zeidan A.M., Pronovost P.J., Haut E.R. (2015). Eliminating healthcare disparities via mandatory clinical decision support: The venous thromboembolism (VTE) example. Med. Care.

[51] Bachhuber M.A., O’Grady M.A., Chung H., Neighbors C.J., DeLuca J., D’Aloia E.M., Diaz A., Cunningham C.O. (2017). Delivery of screening and brief intervention for unhealthy alcohol use in an urban academic Federally Qualified Health Center. Addict. Sci. Clin. Pract..

[52] Bradley K.A., Boyd-Wickizer J., Powell S.H., Burman M.L. (1998). Alcohol screening questionnaires in women: A critical review. JAMA.

[53] Minian N., Baliunas D., Zawertailo L., Noormohamed A., Giesbrecht N., Hendershot C.S., Le Foll B., Rehm J., Samokhvalov A., Selby P.L. (2017). Combining alcohol interventions with tobacco addictions treatment in primary care—The COMBAT study: A pragmatic cluster randomized trial. Implement. Sci..

[54] Minian N., Noormohamed A., Zawertailo L., Baliunas D., Giesbrecht N., Le Foll B., Rehm J., Samokhvalov A., Selby P.L. (2018). A method for co-creation of an evidence-based patient workbook to address alcohol use when quitting smoking in primary care: A case study. Res. Involv. Engagem..

[55] Gomez A., Conde A., Santana J., Jorrin A. (2005). Diagnostic usefulness of brief versions of Alcohol Use Disorders Identification Test (AUDIT) for detecting hazardous drinkers in primary care settings. J. Stud. Alcohol.

[56] Heatherton T.F., Kozlowski L.T., Frecker R.C., Rickert W., Robinson J. (1989). Measuring the heaviness of smoking: Using self-reported time to the first cigarette of the day and number of cigarettes smoked per day. Br. J. Addict..

[57] Bush K., Kivlahan D.R., McDonell M.B., Fihn S.D., Bradley K.A. (1998). The AUDIT alcohol consumption questions (AUDIT-C): An effective brief screening test for problem drinking. Arch. Intern. Med..

[58] StataCorp (2015). Stata Statistical Software: Release 14.

[59] Gomel M.K., Wutzke S.E., Hardcastle D.M., Lapsley H., Reznik R.B. (1998). Cost-effectiveness of strategies to market and train primary health care physicians in brief intervention techniques for hazardous alcohol use. Soc. Sci. Med..

[60] Kaner E.F., Heather N., Mcavoy B.R., Lock C.A., Gilvarry E. (1999). Intervention for excessive alcohol consumption in primary health care: Attitudes and practices of English general practitioners. Alcohol Alcohol..

[61] Seale J.P., Shellenberger S., Tillery W.K., Boltri J., Barton B., McCauley M., Vogel R. (2006). Implementing alcohol screening and intervention in a family medicine residency clinic. Subst. Abus..

[62] Weisner C., Schmidt L. (1992). Gender disparities in treatment for alcohol problems. JAMA.

[63] Thom B., Téllez C. (1986). A difficult business: Detecting and managing alcohol problems in general practice. Br. J. Addict..

[64] Nygaard P., Aasland O.G. (2011). Barriers to implementing screening and brief interventions in general practice: Findings from a qualitative study in Norway. Alcohol Alcohol..

[65] Gomberg E. (1988). Alcoholic women in treatment: The question of stigma and age. Alcohol Alcohol..

[66] Brawarsky P., Brooks D., Mucci L., Wood P. (2004). Effect of physician recommendation and patient adherence on rates of colorectal cancer testing. Cancer Detect. Prev..

[67] Sinclair J., Lawson B., Burge F. (2008). Which patients receive on diet and exercise?: Do certain characteristics affect whether they receive such advice?. Can. Fam. Physician.

[68] Bulloch A.G., Williams J.V., Lavorato D.H., Patten S.B. (2016). Trends in binge drinking in Canada from 1996 to 2013: A repeated cross-sectional analysis. CMAJ Open.

[69] Johnston A.D. (2013). Drink: The Intimate Relationship Between Women and Alcohol.

[70] Stockwell T., Wettlaufer A., Vallance K., Chow C., Giesbrecht N., April N., Asbridge M., Callaghan R., Cukier S., Davis-MacNevin P. (2019). Strategies to Reduce Alcohol-Related Harms and Costs in Canada: A Review of Provincial and Territorial Policies.

[71] Brand D.A., Saisana M., Rynn L.A., Pennoni F., Lowenfels A.B. (2007). Comparative analysis of alcohol control policies in 30 countries. PLos Med..

[72] Madureira-Lima J., Galea S. (2018). Alcohol control policies and alcohol consumption: An international comparison of 167 countries. J. Epidemiol. Community Health.

[73] Patra J., Giesbrecht N., Rehm J., Bekmuradov D., Popova S. (2012). Are alcohol prices and taxes an evidence-based approach to reducing alcohol-related harm and promoting public health and safety? A literature review. Contemp. Drug Probl..

[74] Cook P.J. (2007). Paying the Tab: The Costs and Benefits of Alcohol Control.

[75] Elder R.W., Lawrence B., Ferguson A., Naimi T.S., Brewer R.D., Chattopadhyay S.K., Toomey T.L., Fielding J.E., Services T.F.o.C.P. (2010). The effectiveness of tax policy interventions for reducing excessive alcohol consumption and related harms. Am. J. Prev. Med..

[76] Naimi T.S., Brewer R.D., Miller J.W., Okoro C., Mehrotra C. (2007). What do binge drinkers drink?: Implications for alcohol control policy. Am. J. Prev. Med..

[77] Sornpaisarn B., Shield K.D., Rehm J. (2012). Alcohol taxation policy in Thailand: Implications for other low-to middle-income countries. Addiction.

[78] Anderson P., Chisholm D., Fuhr D.C. (2009). Effectiveness and cost-effectiveness of policies and programmes to reduce the harm caused by alcohol. Lancet.

[79] Giesbrecht N., Wettlaufer A., April N., Asbridge M., Cukier S., Mann R., McAllister J., Murie A., Pauley C., Plamondon L. (2013). Strategies to Reduce Alcohol-Related Harms and Costs in Canada: A Comparison of Provincial Policies.

[80] Agardh E., Högberg P., Miller T., Norström T., Österberg E., Ramstedt M., Rossow I., Stockwell T. (2008). Alcohol Monopoly and Public Health: Potential Effects of Privatization of the Swedish Alcohol Retail Monopoly.

[81] Chikritzhs T., Catalano P., Pascal R., Henrickson N. (2007). Predicting Alcohol-Related Harms from Licensed Outlet Density: A Feasibility Study.

[82] Vendetti J., Gmyrek A., Damon D., Singh M., McRee B., Del Boca F. (2017). Screening, brief intervention and referral to treatment (SBIRT): Implementation barriers, facilitators and model migration. Addiction.

[83] Hargraves D., White C., Frederick R., Cinibulk M., Peters M., Young A., Elder N. (2017). Implementing SBIRT (Screening, Brief Intervention and Referral to Treatment) in primary care: Lessons learned from a multi-practice evaluation portfolio. Public Health Rev..

[84] Mertens J.R., Chi F.W., Weisner C.M., Satre D.D., Ross T.B., Allen S., Pating D., Campbell C.I., Lu Y.W., Sterling S.A. (2015). Physician versus non-physician delivery of alcohol screening, brief intervention and referral to treatment in adult primary care: The ADVISe cluster randomized controlled implementation trial. Addict. Sci. Clin. Pract..

[85] Muench J., Jarvis K., Vandersloot D., Hayes M., Nash W., Hardman J., Grover P., Winkle J. (2015). Perceptions of clinical team members toward implementation of SBIRT processes. Alcohol. Treat. Q..

[86] Greaves L., Poole N. (2004). Victimized or validated? Responses to substance-using pregnant women. Can. Woman Stud..

[87] Jacobs L. (2014). ‘Bad’ mothers have alcohol use disorder: Moral panic or brief intervention?. Gend. Behav..

[88] Nathoo T. (2018). Doorways to Conversation: Brief Intervention on Substance Use With Girls and Women.

[89] Wagner E., Babaei M. (2019). Provincial Guideline for the Clinical Management of High-Risk Drinking and Alcohol Use Disorder.

[90] Tannenbaum C., Greaves L., Graham I.D. (2016). Why sex and gender matter in implementation research. Bmc Med. Res. Methodol..

